# Loss of the Bacterial Flagellar Motor Switch Complex upon Cell Lysis

**DOI:** 10.1128/mBio.00298-21

**Published:** 2021-06-08

**Authors:** Mohammed Kaplan, Elitza I. Tocheva, Ariane Briegel, Megan J. Dobro, Yi-Wei Chang, Poorna Subramanian, Alasdair W. McDowall, Morgan Beeby, Grant J. Jensen

**Affiliations:** a Division of Biology and Biological Engineering, California Institute of Technology, Pasadena, California, USA; b Department of Chemistry and Biochemistry, Brigham Young University, Provo, Utah, USA; National Institute of Child Health and Human Development (NICHD)

**Keywords:** cell lysis, flagellar motor, cryo-ET, switch complex

## Abstract

The bacterial flagellar motor is a complex macromolecular machine whose function and self-assembly present a fascinating puzzle for structural biologists. Here, we report that in diverse bacterial species, cell lysis leads to loss of the cytoplasmic switch complex and associated ATPase before other components of the motor. This loss may be prevented by the formation of a cytoplasmic vesicle around the complex. These observations suggest a relatively loose association of the switch complex with the rest of the flagellar machinery.

## OBSERVATION

The bacterial flagellar motor is a complex nanomachine responsible for cell motility in a wide range of species. It consists of a cell envelope-embedded motor that rotates an extracellular filament, connected by a universal joint known as the hook (1). The motor is composed of a stator consisting of ion channels embedded in the cytoplasmic or inner membrane (IM) and a rotor consisting of a cytoplasmic switch complex (also called the C-ring), a membrane/supramembrane (MS)-ring, a periplasmic driveshaft known as the rod, associated bushings in the peptidoglycan cell wall (the P-ring), and, in diderms, the outer membrane (the lipopolysaccharide, or L-ring) (1). A flagellar type III secretion system (fT3SS) is responsible for self-assembly of the machine, which begins with the IM-associated components and proceeds in a stepwise fashion outward to the extracellular ones (1). In addition to this conserved core, the flagella of various species can have periplasmic or extracellular species-specific components that adorn their flagella (2, 3).

The flagellar machinery can also disassemble, as in the programmed ejection of the flagellum during the life cycle of the alphaproteobacterium Caulobacter crescentus. This process is thought to be accompanied by the digestion of the C terminus of the MS-ring protein FliF (4). More recently, flagellar disassembly has been observed in many species under starvation or mechanical stress. This process starts with loss of flagellar hooks and filaments and continues with disassembly of motor components, leaving plugged P- and L-rings in the cell wall and outer membrane (5–11). Additionally, we have recently shown that programmed flagellar ejection in *C. crescentus* leaves similar plugged P- and L-rings suggesting an evolutionary link between this process and the starvation-induced one (12).

**FIG 1:**
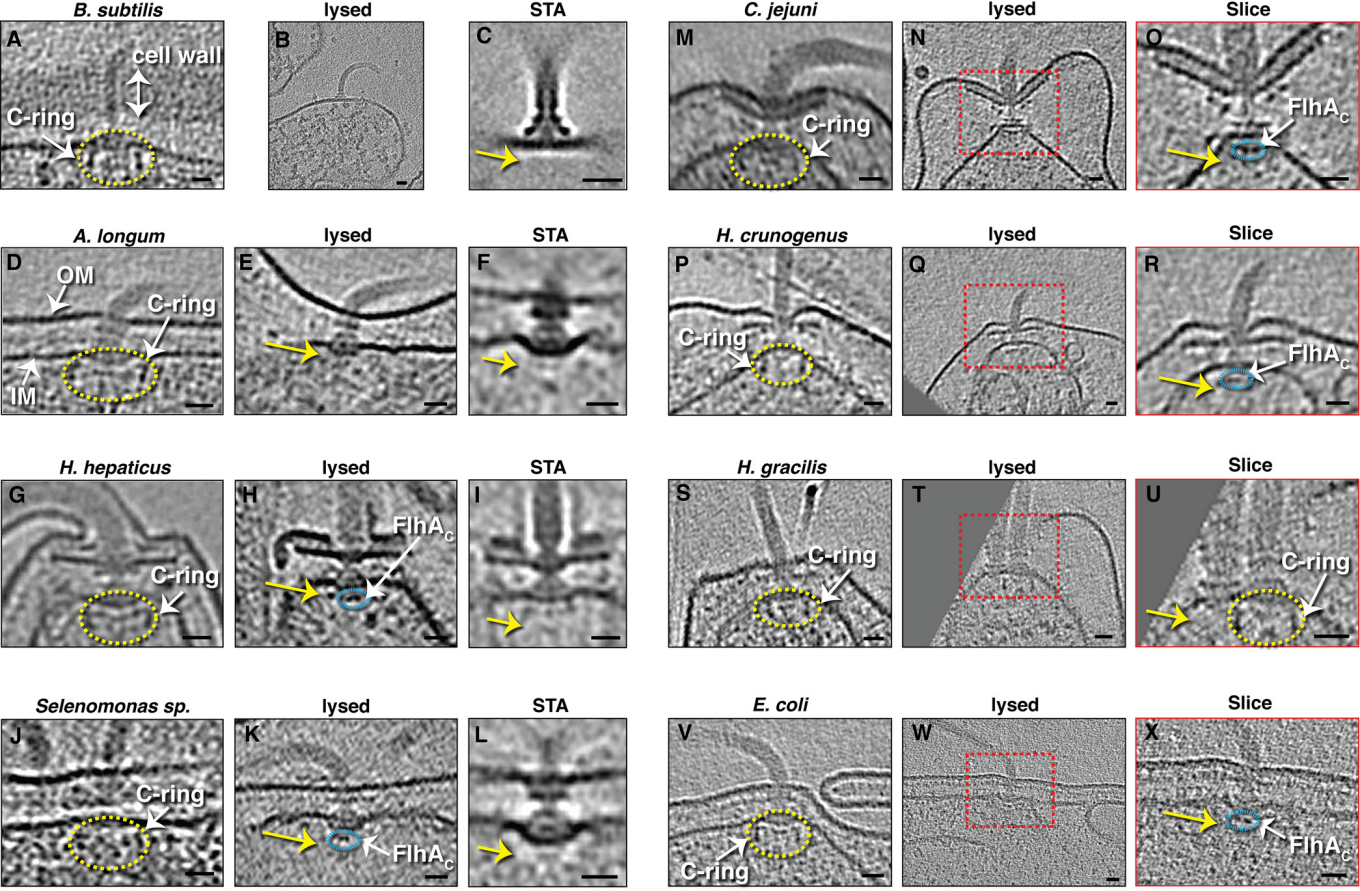
Slices through electron cryotomograms (except panels C, F, I, and L) of intact or lysed cells of various species showing flagella either with the switch complex (yellow ellipses) or lacking the switch complex (yellow arrows point to the expected position of the absent C-ring). Cyan ellipses indicate densities belonging to FlhA_C_. Red boxes in panels N, Q, T, and W indicate regions enlarged in the panel to the right. (C, F, I, and L) Subtomogram averages (STA) of the subcomplexes shown in panels B, E, H, and K, respectively. Scale bars, 20 nm. IM, inner membrane; OM, outer membrane.

The complexity of the flagellar motor and its location spanning the cell envelope mean that it is challenging to purify intact. The development of cryogenic electron tomography (cryo-ET), the highest-resolution imaging technique currently applicable to unique biological objects, has enabled the structure to be studied *in situ* in a native state inside cells. Over the past 15 years, our laboratory has collected tens of thousands of electron cryotomograms of dozens of bacterial species (13). This resource includes many examples of lysed cells, collected either accidentally or intentionally. For example, we have found that lysis of Escherichia coli cells by light penicillin treatment flattens them, making them more suitable for cryo-ET experiments (14). Similarly, the thick cell wall of Bacillus subtilis renders them a challenging sample for cryo-ET experiments, so we digested the cell wall of B. subtilis with lysozyme to visualize membrane-embedded structures in protoplasts and cell lysates. While examining these lysates, we identified a flagellar subcomplex that lacks the C-ring and contains only the MS-ring, the rod, and the hook (occasionally with part of the filament) (Fig. 1A and B). We identified a total of 71 such particles in 26 cryotomograms, enabling us to calculate a subtomogram average of the structure (Fig. 1C).

Wondering whether C-ring loss after cell lysis is a general phenomenon, we examined electron cryotomograms of lysed cells from different phyla (*Proteobacteria* and *Firmicutes*) available in our database. Besides the results in B. subtilis, we identified lysed cells retaining flagellar filaments in E. coli (penicillin treated), Helicobacter hepaticus, Campylobacter jejuni, Hydrogenovibrio crunogenus, Hylemonella gracilis, *Selenomonas* sp. (clinical isolate), and Acetonema longum. In each case, in addition to fully intact flagella (which have the switch complex), we also observed flagellar filaments and hooks connected to motors lacking the C-ring (Fig. 1D to X). Multiple examples were found in some species that allowed us to average them (H. hepaticus, 7 examples in 7 lysed cells; A. longum, 7 examples in 2 lysed cells; *Selenomonas* sp., 6 examples in 2 lysed cells) (Fig. 1F, I, and L). In roughly half of these partially disassembled motors, we observed densities corresponding to the C terminus of the fT3SS protein FlhA (FlhA_C_) at the base of the MS-ring (Fig. 1H, K, O, R, and X, cyan circles).

**FIG 2:**
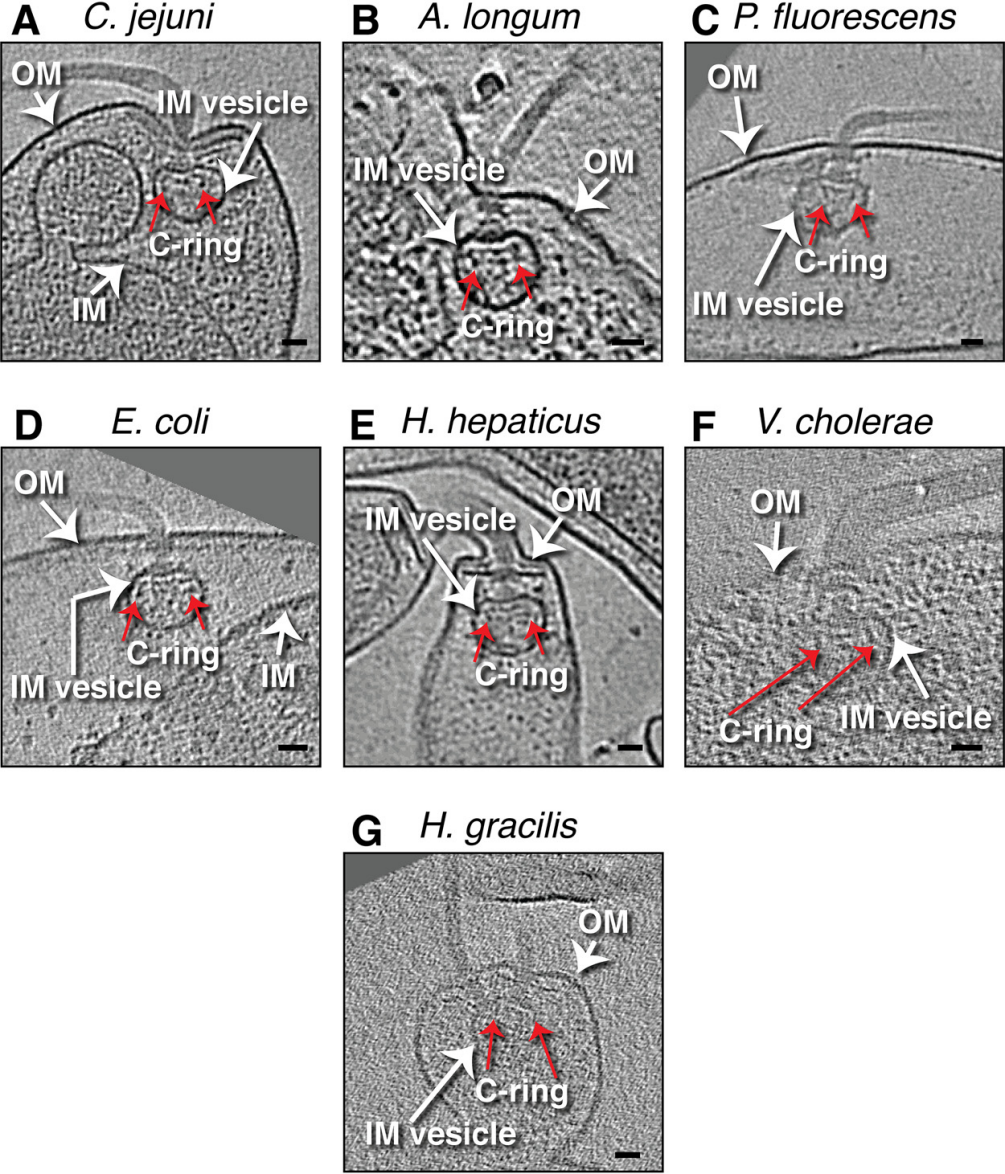
(A to G) Slices through electron cryotomograms of lysed cells of the indicated species, highlighting the presence of a flagellum with an intact C-ring (red arrows) encapsulated by an IM vesicle. Scale bars, 25 nm.

Less frequently, lysed cells contained a small vesicle of IM encapsulating the cytoplasmic components of the flagellar motor (C. jejuni, 2 examples in 2 lysed cells; A. longum, 4 examples in 4 lysed cells; H. hepaticus, 4 examples in 4 lysed cells; single examples for the rest). These vesicles were only slightly larger than the switch complex, and, in each case, the C-ring was retained (Fig. 2).

**FIG 3:**
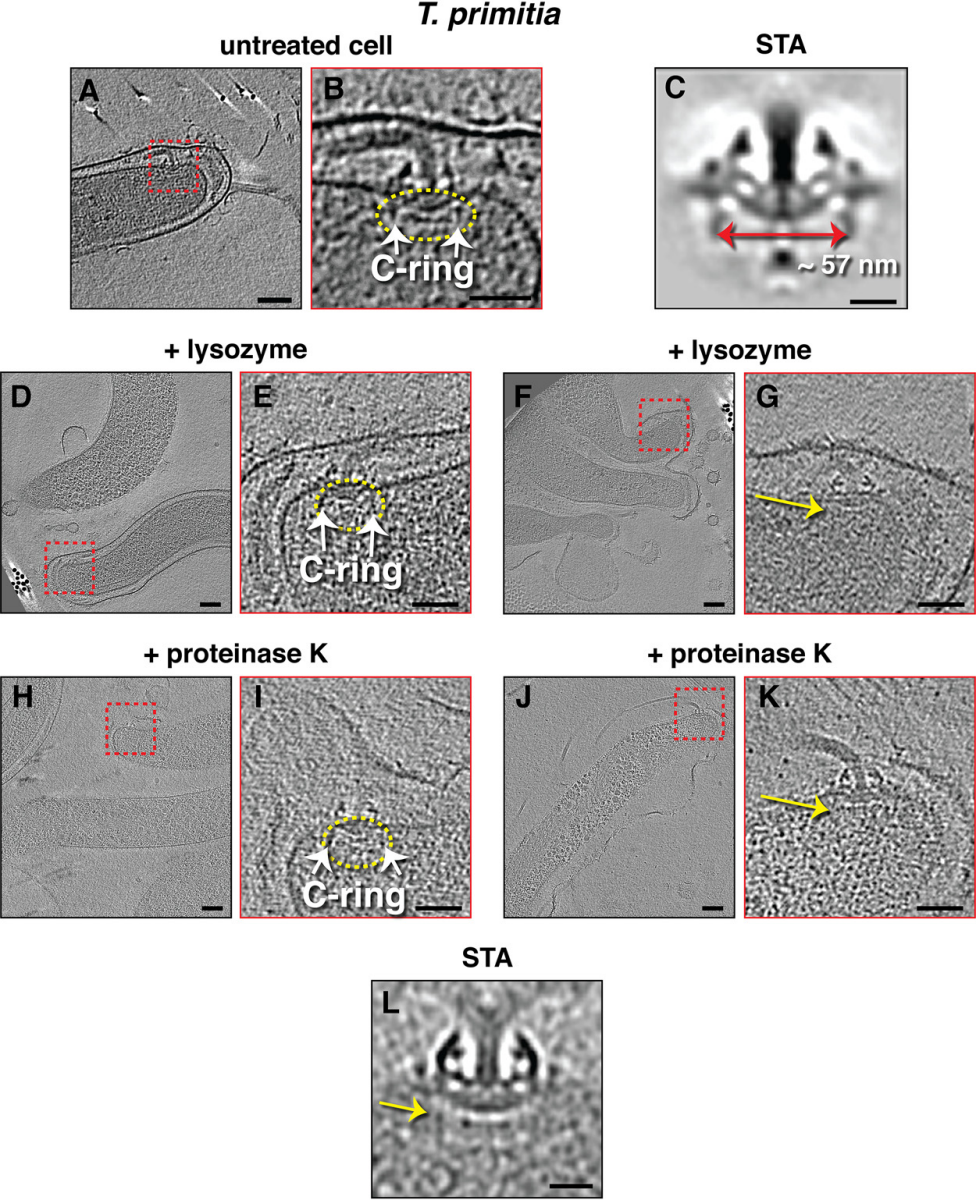
Slices through electron cryotomograms of undigested (A and B), lysozyme-digested (D to G), or proteinase K-digested (H to K) *T. primitia* cells. Red boxes in panels A, D, F, H, and J indicate regions enlarged in the panel to the right. Flagella with or without the switch complex are indicated by yellow ellipses or yellow arrows (pointing to the expected position of the absent C-ring), respectively. Scale bars in panels A, D, F, H, and J, 100 nm; in panels B, E, G, I, and K, 50 nm. (C and L) Subtomogram averages (STA) of an intact flagellar motor (EMD-1235; see reference 31) (C) or a motor lacking the switch complex present in digested cells (L). Scale bars, 20 nm.

As the cases mentioned above were either from complete cell lysates (as in B. subtilis), partially lysed cells (E. coli), or cells that were randomly lysed during sample preparation (the rest of the examples), we investigated this correlation between cell lysis and the loss of the switch complex by imaging cells that have undergone a more controlled lysis. To that end, we prepared and imaged Treponema primitia cells, which are spirochetes with periplasmic flagella, treated with different digestive enzymes: (i) cells incubated with 5 mg/ml lysozyme for 15 min prior to plunge-freezing, (ii) cells incubated with 5 mg/ml proteinase K for 15 min prior to plunge freezing, and (iii) undigested cells as a control group. The cells in groups 1 and 2 lacked an intact outer membrane and cell wall (Fig. 3). While the 25 motors identified in untreated cells all had C-rings, 6 of the 16 motors present in cells treated with lysozyme lacked the C-ring, and 1 motor out of the 9 motors in cells treated with proteinase K lacked the C-ring (Table 1, Fig. 3), suggesting again a correlation between cell lysis and C-ring loss.

**TABLE 1:** Number of motors with or without C-ring in digested or untreated *T. primitia*

Treponema primitia	No. of motors with C-ring	No. of motors without C-ring	No. unclear	No. of tomograms
Untreated cells	25	0	0	18
+Lysozyme	8	6	2	13
+Proteinase K	8	1	0	5

These results suggest that, upon cell lysis, the C-ring of some motors dissociates from the MS-ring but is retained if the proteins are encapsulated by a vesicle of cytoplasm. It is possible that the higher protease activity in the cytoplasm compared to the periplasm (15) plays a role in the loss of the cytoplasmic parts of the motor (i.e., the C-ring and the ATPase), but the preservation of the cytoplasmic FlhA_C_ suggests dissociation of the C-ring rather than proteolysis. This leads us to conclude that the interaction between the C- and MS-rings is more labile than interactions between other flagellar components. This also would explain the absence of the switch complex in early preparations of purified flagella (16, 17). Later studies found that the switch complex can be retained if flagella are purified under mild conditions of pH and salt (18–21), but even under mild conditions, the majority of purified flagella lose their C-rings after 20 minutes at room temperature or 80 hours at 4°C (20).

In our case, the lysis conditions were different for the various samples. For example, B. subtilis was completely lysed with lysozyme prior to plunge-freezing and imaging, and only cell lysate was present in the cryotomograms, while E. coli was mildly lysed by incubating the sample with penicillin before plunge-freezing. On the other hand, *T. primitia* was mildly lysed with lysozyme and proteinase K. The majority of the observed particles lacked the C-ring in the lysate of B. subtilis, but only a small proportion of motors were devoid of the switch complex in mildly lysed E. coli and *T. primitia*. While this could be related to the different lysis conditions and how severely the cells are lysed, we cannot exclude species-specific differences in this process. As for other species, the examples we identified were randomly lysed during sample preparation and we anticipate that the lysis conditions were mild (standard growth media), but we do not know how long individual cells were lysed before plunge-freezing. Whereas these results suggest that the loss of the switch complex is more prominent under severe lysis (like in B. subtilis), further experiments are required to quantify this process in relation to different lysis conditions (including mechanical stress) in different species.

In the case of partially lysed *T. primitia*, only the outer membrane and cell wall appeared damaged, while the cells continued to have an intact inner membrane and normal cytoplasm, as seen in our cryotomograms. It is known that the stators interact with the cell wall upon incorporation into the flagellar motor to get activated (22), and the damage of the cell wall in these cells could interfere with the incorporation of stators to the motor and their interaction with the switch complex. The loss of C-ring in these cells could be due to the interruption of this interaction, which would normally help to stabilize the C-ring in its place.

Previous studies of flagellar loss in intact Vibrio alginolyticus cells suggest that this process starts with the detachment, not digestion, of the C-ring, which is freed to diffuse along the inner membrane (9), and intact C-rings do not copurify with the motor in this species (23). Cryo-ET of intact Shewanella oneidensis cells lacking the filament proteins FlaA and FlaB (Δ*flaAB* mutant) also revealed a flagellar subcomplex with the extracellular hook and periplasmic components but without the cytoplasmic ones (6), further suggesting that C-ring loss is not limited to cell lysis. It is conceivable that releasing the C-ring is beneficial to cells in order to save energy when there is no filament to rotate, as in the S. oneidensis Δ*flaAB* mutant.

The phenomenon of breaking the bacterial flagellum and leaving stable flagellar subcomplexes under certain conditions is a recently described one. First, we and others showed that various bacteria can lose their flagella under starvation, leaving the P- and L-rings as a stable subcomplex in the outer membrane (5–11). However, how the motor breaks to leave PL-rings remains unknown. Using fluorescence microscopy, Zhuang et al. presented a model of the possibility of breaking the bacterial flagellum at the rod level, leaving the MS- and C-rings detached (9). While we have no evidence that our observations in the current work are connected to the starvation-related process, they add to the repertoire of stable flagellar subcomplexes under stress conditions, where the C-ring dissociates from the motor without the MS-ring leaving the rest of the flagellum (including the extracellular parts) as a stable structure.

The evolution of the bacterial flagellum is believed to have started as a primordial secretion system that subsequently added the periplasmic and extracellular components (rod, hook, and filament) by multiple gene diversification and duplication events (24). This connection between the flagellum and a secretion system is further bolstered by its structural similarity to the bacterial type III secretion system (25, 26), where it is believed that the evolution of the flagellum preceded that of the type III secretion system (27). However, a recent high-resolution structure of purified flagellar basal bodies highlighted structural differences between these two molecular machines (28). Presumably, tinkering of a primordial secretion system with ion channels and the addition of the switch complex led to the formation of the first motor. However, was the C-ring added before or after the union between the preliminary secretion system and the ion channels? When did the rod, hook, and filament evolve? While both the preliminary secretion system and ion channels could have had independent functions unrelated to cell motility, it remains unknown what the role of the ancestral C-ring proteins was before functioning as a switch complex. Just as the secretion and assembly of the rod, hook, and filament proteins mirror their evolutionary past (24), it could be that the flagellar relics lacking the switch complex identified here, and the PL-subcomplexes in the starvation-related process, also reflect major modular transitions in the history of flagellar evolution.

### Experimental procedures.

Cell growth, sample preparation, cryo-ET imaging, and image processing were performed as previously described for E. coli and H. hepaticus (14), *H. crunogenus* (29), A. longum (30), *T. primitia* (31), and Vibrio cholerae (32). Pseudomonas fluorescens was grown in K10 medium as described in reference 33 and C. jejuni as described in reference 34. *H. gracilis* was grown for 48 h in broth 233 at 26°C without antibiotics to a final optical density at 600 nm of <0.1 and subsequently incubated with attack-phase Bdellovibrio bacteriovorus for 3 days, after which cells were spun down at 1,000 × *g* for 5 min and concentrated ∼10× for plunge-freezing. *Selenomonas* spp. were isolated from the human gut by Emma Allen-Vercoe, University of Guelph. Cells were grown anaerobically in fastidious anaerobe agar supplemented with 5% defibrinated sheep’s blood. B. subtilis protoplasts (obtained from the D. Kearns laboratory, Indiana University) were prepared with lysozyme using a modified protocol based on reference 35. Subsequent imaging and processing were performed as described for other species. Subtomogram averaging was done as in reference 6.
